# Bioinformatics analysis identifies a key gene HLA_DPA1 in severe influenza-associated immune infiltration

**DOI:** 10.1186/s12864-024-10184-7

**Published:** 2024-03-07

**Authors:** Liang Chen, Jie Hua, Xiaopu He

**Affiliations:** 1 Department of Infectious Diseases, Taikang Xianlin Drum Tower Hospital, Affiliated Hospital of Medical College of Nanjing University, No 188, Lingshan North Road, Qixia District, Nanjing, 210046 China; 2 Department of Gastroenterology, The First Affiliated Hospital of Nanjing Medical University, Nanjing, China; 3 Department of Geriatric Gastroenterology, The First Affiliated Hospital of Nanjing Medical University, Nanjing, China

**Keywords:** Severe influenza, Invasive mechanical ventilation, HLA_DPA1, Bioinformatics analysis

## Abstract

**Background:**

Severe influenza is a serious global health issue that leads to prolonged hospitalization and mortality on a significant scale. The pathogenesis of this infectious disease is poorly understood. Therefore, this study aimed to identify the key genes associated with severe influenza patients necessitating invasive mechanical ventilation.

**Methods:**

The current study utilized two publicly accessible gene expression profiles (GSE111368 and GSE21802) from the Gene Expression Omnibus database. The research focused on identifying the genes exhibiting differential expression between severe and non-severe influenza patients. We employed three machine learning algorithms, namely the Least Absolute Shrinkage and Selection Operator regression model, Random Forest, and Support Vector Machine-Recursive Feature Elimination, to detect potential key genes. The key gene was further selected based on the diagnostic performance of the target genes substantiated in the dataset GSE101702. A single-sample gene set enrichment analysis algorithm was applied to evaluate the participation of immune cell infiltration and their associations with key genes.

**Results:**

A total of 44 differentially expressed genes were recognized; among them, we focused on 10 common genes, namely PCOLCE2, HLA_DPA1, LOC653061, TDRD9, MPO, HLA_DQA1, MAOA, S100P, RAP1GAP, and CA1. To ensure the robustness of our findings, we employed overlapping LASSO regression, Random Forest, and SVM-RFE algorithms. By utilizing these algorithms, we were able to pinpoint the aforementioned 10 genes as potential biomarkers for distinguishing between both cases of influenza (severe and non-severe). However, the gene HLA_DPA1 has been recognized as a crucial factor in the pathological condition of severe influenza. Notably, the validation dataset revealed that this gene exhibited the highest area under the receiver operating characteristic curve, with a value of 0.891. The use of single-sample gene set enrichment analysis has provided valuable insights into the immune responses of patients afflicted with severe influenza that have further revealed a categorical correlation between the expression of HLA_DPA1 and lymphocytes.

**Conclusion:**

The findings indicated that the HLA_DPA1 gene may play a crucial role in the immune-pathological condition of severe influenza and could serve as a promising therapeutic target for patients infected with severe influenza.

**Supplementary Information:**

The online version contains supplementary material available at 10.1186/s12864-024-10184-7.

## Background

Despite advances in biomedicine, the incidence of hospitalization and mortality rates elicited by influenza, a profoundly contagious respiratory disease, persistently exhibit an upward trend [1, 2]. The global prevalence of symptomatic flu is estimated to range from 10 to 20% annually, affecting a substantial portion of the population. However, the chronic manifestation of this disease afflicts approximately 3–5 million individuals worldwide. Tragically, influenza-related mortality rates vary between 290,000 and 650,000 deaths [3]. Furthermore, the clinical manifestations of influenza encompass a diverse array of symptoms, from acute upper respiratory tract infections to the development of severe pneumonia [4]. Conversely, some patients afflicted with severe influenza frequently exhibit respiratory dysfunction, as evidenced by reduced arterial pressure of oxygen to a fraction of the inspired oxygen ratio (≦ 200 mmHg). Consequently, these patients rely on IMV for respiratory support, and critical patients’ death rate reaches about 50–80% [5, 6]. The detailed mechanisms governing the pathological condition of severe influenza remain elusive.

Previous studies reported that immune cells and pathways are pivotal to the occurrence and progression of severe influenza [7, 8]. Reliable immunological biomarkers are urgently required to prevent and treat patients with severe influenza infections. Microarray technologies and bioinformatic analyses have been widely used to identify disease-specific biomarkers [7, 9]. However, due to the presence of sample heterogeneity and variations in sampling methods, as well as the utilization of diverse technology platforms and analysis strategies across individual studies, the execution of statistical analyses and the extraction of esteemed information pose significant challenges.

**Fig. 1:**
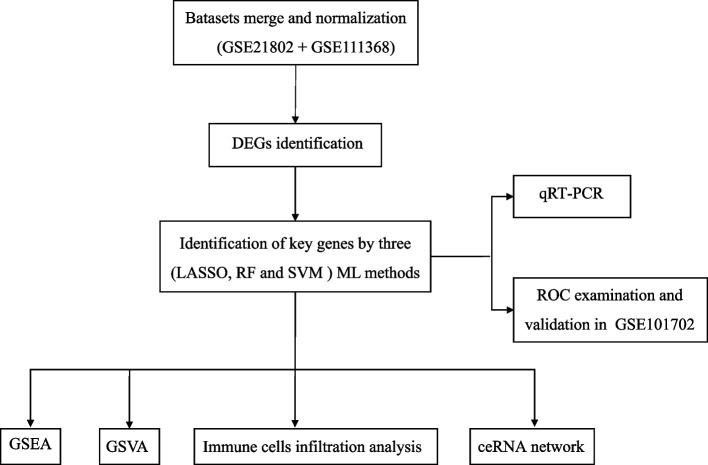
The study flow chart

Hence, the integration of bioinformatics approaches together with expression profiling techniques presents an opportunity to obtain a comprehensive understanding of the molecular mechanisms underlying influenza infection. This approach can yield valuable insights and facilitate the development of novel molecular signatures. Here, we elucidated the key genes implicated in the requirement of IMV among influenza patients through bioinformatics analysis. Additionally, we sought to investigate the association among these genes and the levels of infiltrating distinct immune cells. The study design can be seen in Fig. 1.

## Results

### Cross‐platform normalization

**Fig. 2:**
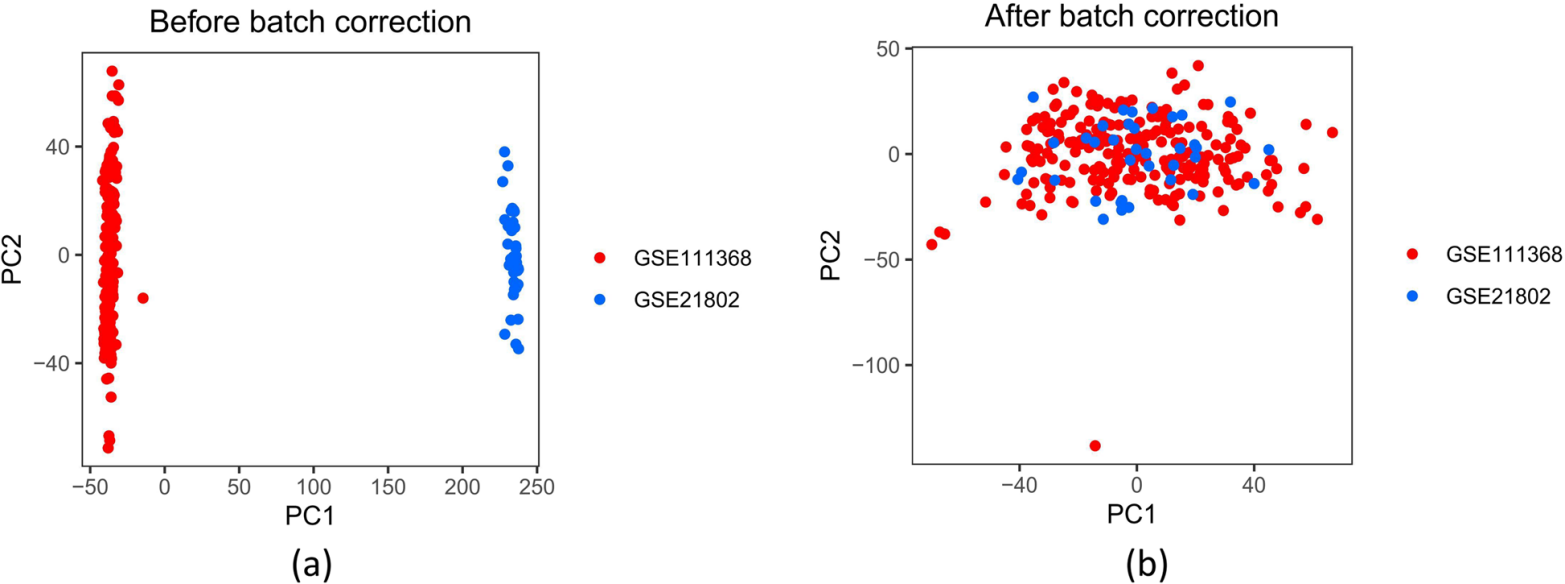
Principal component analysis of gene expression data set. The dots in the scatter plot are based on the first two main components of the gene expression profile (PC1 and PC2) visualization samples: **a** no elimination of batch effect; **b** elimination of batch effect. The colors represent samples from two different data sets

The microarray platforms collectively identified 12,031 genes in the two patient samples. Before applying batch-effect removal techniques, the samples displayed clustering patterns influenced by batch effects along the two principal component (PC) axes with the highest variance. These axes were determined using gene expression values that had not been normalized (Fig. 2a). The principal component analysis (PCA) analysis conducted after normalization has validated the effective removal of batch effects (Fig. 2b), demonstrating the successful implementation of cross-platform normalization.

### DEGs identification and functional analyses

**Fig. 3:**
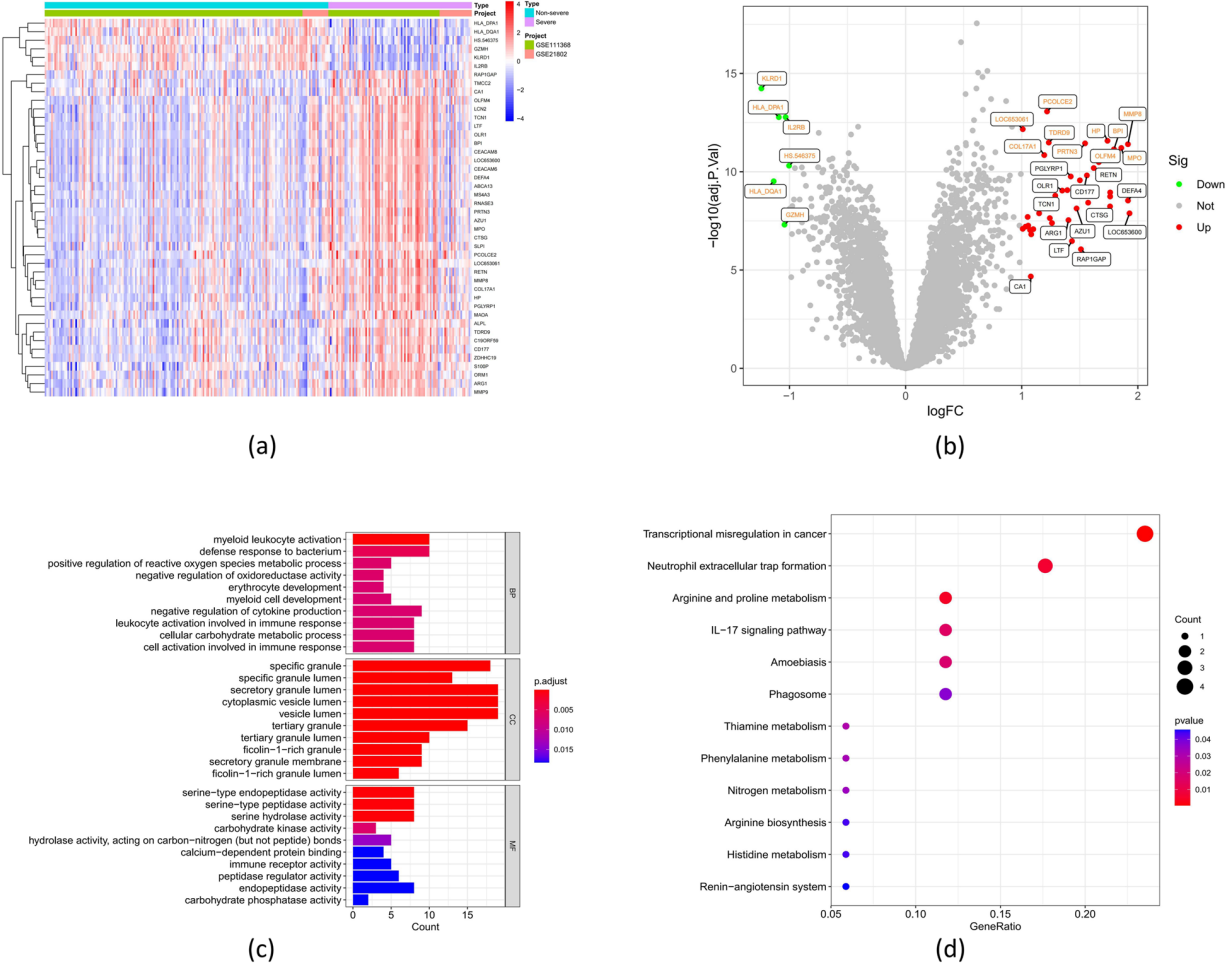
Expression levels of differentially expressed genes (DEGs) in samples of severe and non-severe influenza. **a** Heatmap showing expression patterns of DEGs. **b** Map of DEGs. Upregulated genes are marked in light red; downregulated genes are marked in light green; the top and bottom 10 genes are marked in yellow. The enrichment analysis for DEGs results of GO (**c**) and KEGG (**d**) pathway. Adjusted *P*-value < 0.05 was considered significant (Fisher test)

A total of 44 DEGs between both types of influenza (severe and non-severe) samples were identified from the training dataset, including six downregulated DEGs and 38 upregulated DEGs (Fig. 3a, b). GO and KEGG enrichment analyses were employed to elucidate the specific biological roles played by the DEGs in severe influenza. GO analysis suggested that the DEGs were associated with the process of myeloid leukocyte activation, defence response to bacteria, and regulation of cytokine production (Fig. 3c and Supplementary File 1). KEGG enrichment analysis exhibited that the DEGs were predominantly involved in the pathways of transcriptional misregulation in cancer, neutrophil extracellular trap formation, and the IL − 17 signaling pathway (Fig. 3d and Supplementary File 2). In summary, the DEGs were mainly involved in immune and inflammatory responses.

### Identification of the key gene for severe influenza

**Fig. 4:**
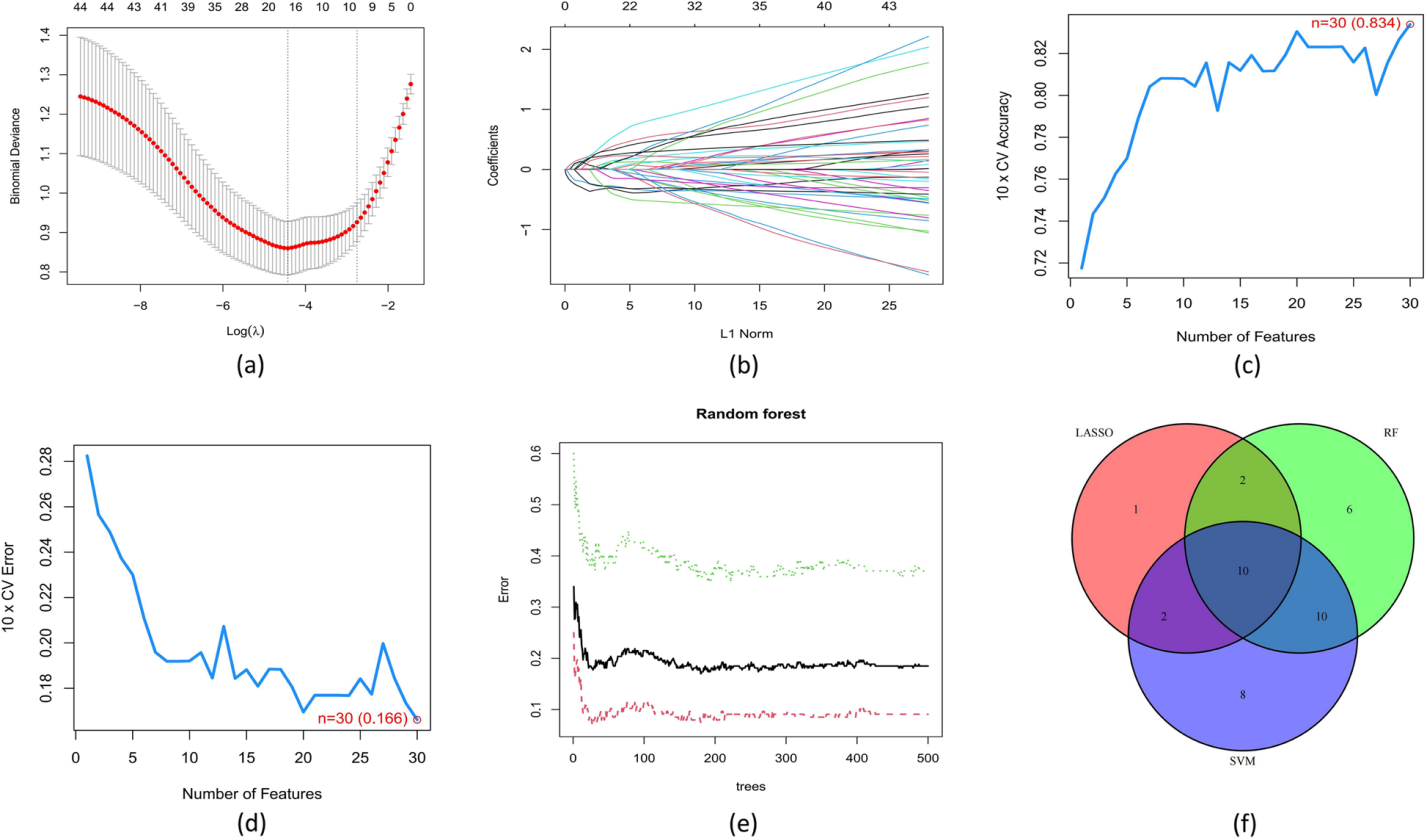
Identification of candidate key genes for severe influenza by three machine-learning algorithms: Least Absolute Shrinkage and Selection Operator (LASSO) regression (**a**, **b**), Support Vector Machine-Recursive Feature Elimination (SVM-RFE) (**c**, **d**), and Random Forest (RF) (**e**, **f**). **g** The overlapping genes of the three algorithms were identified as the candidate key genes for severe influenza

**Fig. 5:**
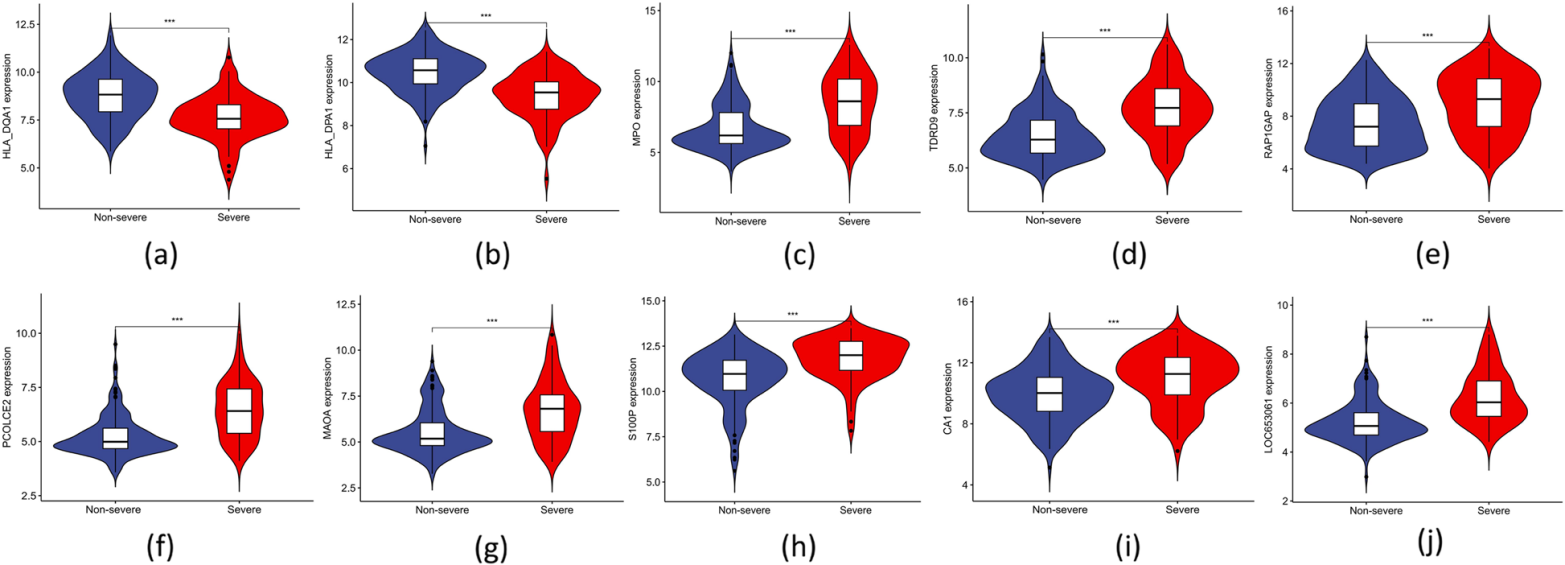
The expression level of the candidate key genes, **a** HLA_DQA1, **b** HLA_DPA1, **c** MPO, **d** TDRD9, **e** RAP1GAP, **f** PCOLCE2, **g** MAOA, **h** S100P, **i** CA1, **j** LOC653061, in the training cohort. *p < 0.05; **p < 0.01; ***p < 0.001

**Fig. 6:**
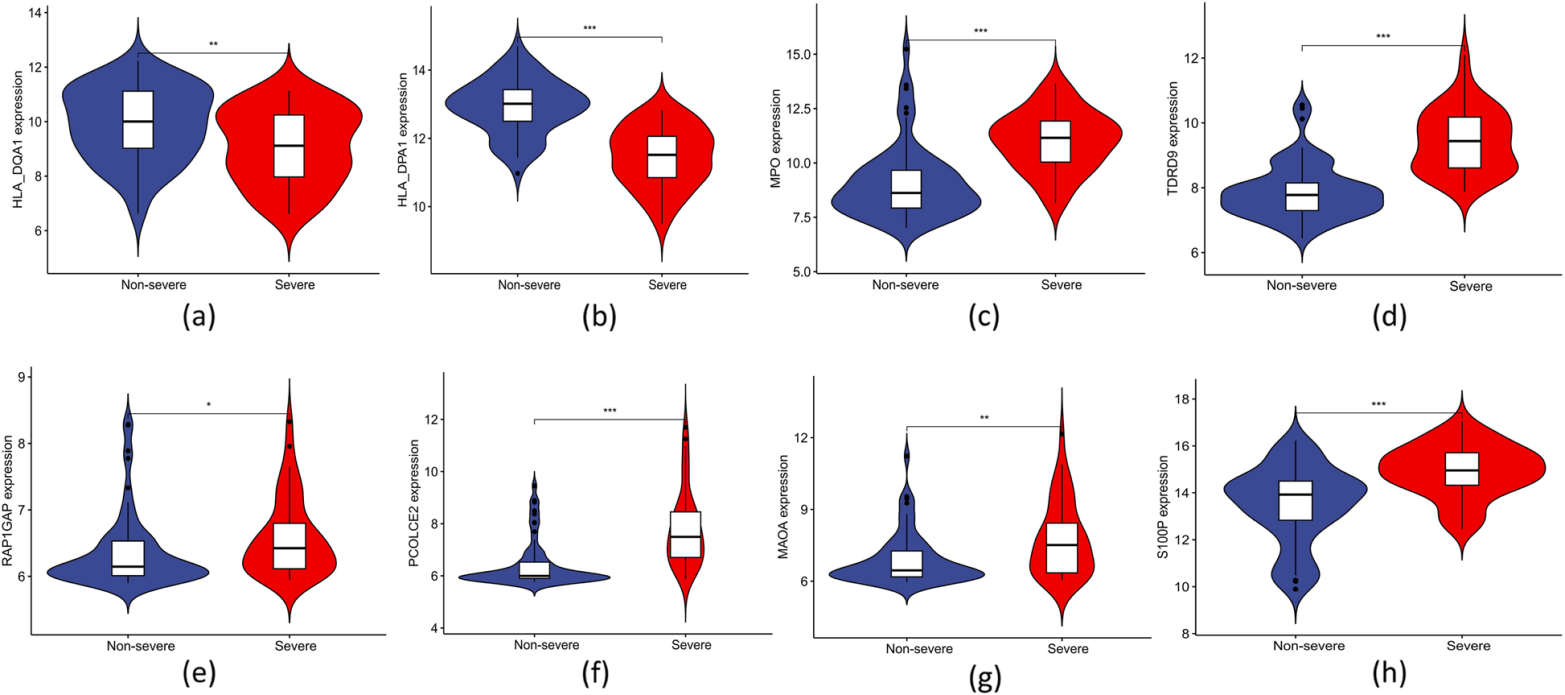
The expression level of the candidate key genes, **a** HLA_DQA1, **b** HLA_DPA1, **c** MPO, **d** TDRD9, **e** RAP1GAP, **f** PCOLCE2, **g** MAOA, **h** S100P, in the validation cohort. *p < 0.05; **p < 0.01; ***p < 0.001

**Fig. 7:**
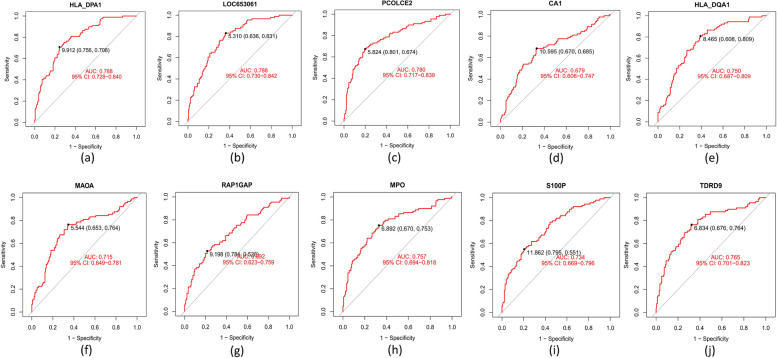
The ROC curves of the candidate key genes, **a** HLA_DQA1, **b** LOC653061, **c** PCOLCE2, **d** CA1, **e** HLA_DQA1, **f** MAOA, **g** RAP1GAP, **h** MPO, **i** S100P, **j** TDRD9, in the training dataset

**Fig. 8:**
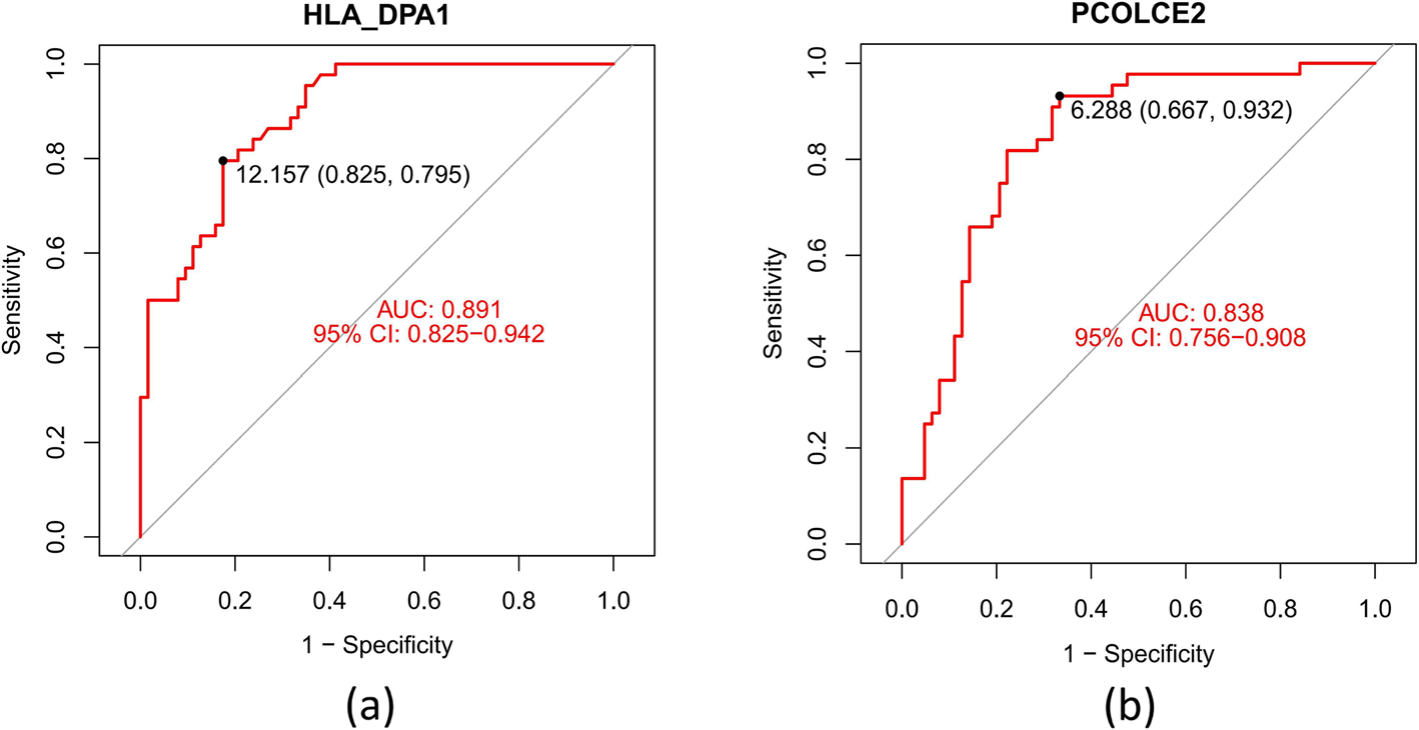
The ROC curves of the candidate key genes in the validation dataset. Only HLA_DPA1 (**a**) and PCOLCE2 (**b**) had a AUC above 0.7

The ten common DEGs (PCOLCE2, HLA_DPA1, LOC653061, TDRD9, MPO, HLA_DQA1, MAOA, S100P, RAP1GAP, and CA1) that were obtained by overlapping genes from computing the three algorithms [LASSO regression (Fig. 4a, b), SVM-RFE algorithms (Fig. 4c, d), and RF (Fig. 4e, f) are candidate key genes for severe influenza (Fig. 4g). HLA_DPA1 and HLA_DQA1 expression was significantly lower in patients with severe influenza in the training dataset compared to patients with non-severe influenza. In contrast, PCOLCE2, TDRD9, MPO, MAOA, RAP1GAP, and S100P expression was higher in the severe influenza group compared to the non-severe influenza group in the training cohort (Fig. 5a-h), similar to the findings in the validation cohort (Fig. 6a-h). The expression of LOC653061 and CA1 was greater in severe influenza patients in contrast to non-severe patients in the training dataset (Fig. 5i, j), whereas it was comparable in the validation dataset. In the training dataset, HLA_DPA1 and LOC653061 genes exhibited the highest AUC of 0.788 as depicted in Fig. 7a, b, while others were below 0.7 (Fig. 7c-j). Conversely, in the validation dataset, the AUCs of HLA_DPA1 and PCOLCE2 were 0.891 and 0.838, respectively (Fig. 8a, b). The AUCs of all eight candidate genes were found to be less than 0.7. Thus, HLA_DPA1 was selected as a key gene in patients diagnosed with severe influenza needing IMV.

**Fig. 9:**
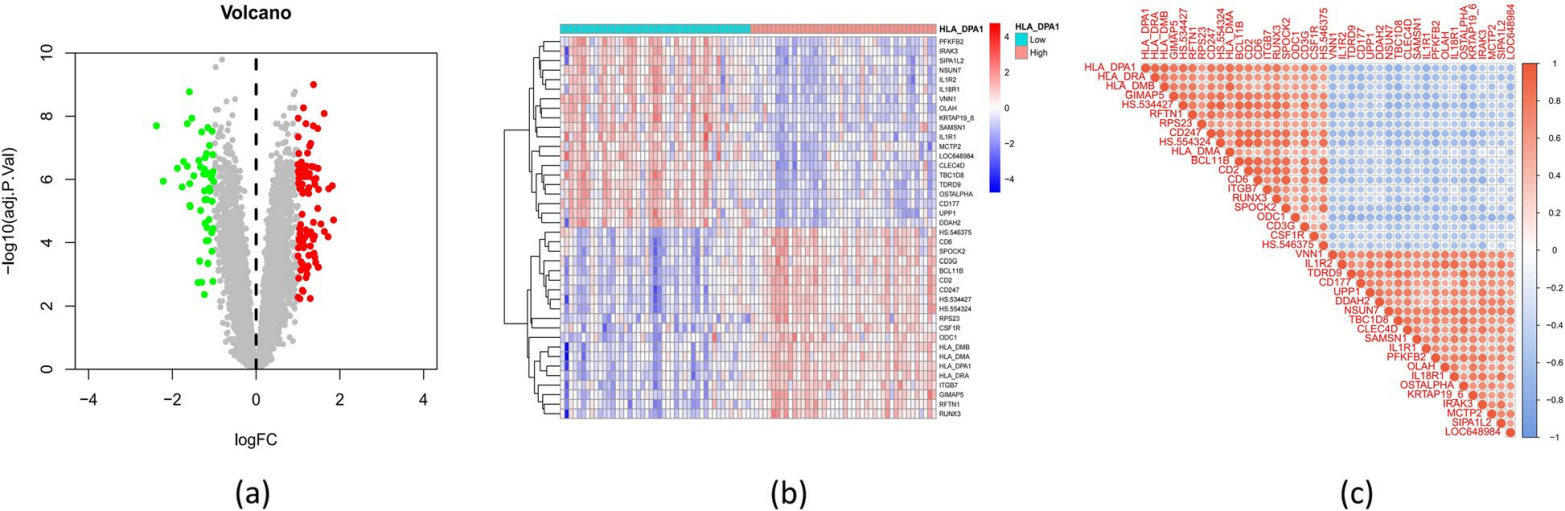
Two groups based on the median value of HLA_DPA1 expression. The volcano map (**a**) and heatmap (**b**) of expression patterns of genes between HLA_DPA1high and HLA_DPA1low groups. Upregulated genes are marked in light red; downregulated genes are marked in light green. **c** The Pearson correlation of these genes

The severe influenza samples were categorized into two distinct groups by employing a division based on the median value of HLA_DPA1 expression: HLA_DPA1^low^ (*n* = 44) and HLA_DPA1^high^ (*n* = 45). Some genes (e.g., SPOCK2, ITGB7, GIMAP5, et al.) were upregulated, while others (e.g., PFKFB2, IRAK3, SIPA1L2, et al.) were downregulated in the group of HLA_DPA1^high^ (Fig. 9a, b). The correlation between HLA_DPA1 and the other genes in the training dataset is shown in Fig. 9c. The median expression level of HLA_DPA1 from the training dataset in severe and non-severe influenza patients was 9.540 and 10.572, respectively.

### Identification of the key gene via GSEA and GSVA analyses

**Fig. 10:**
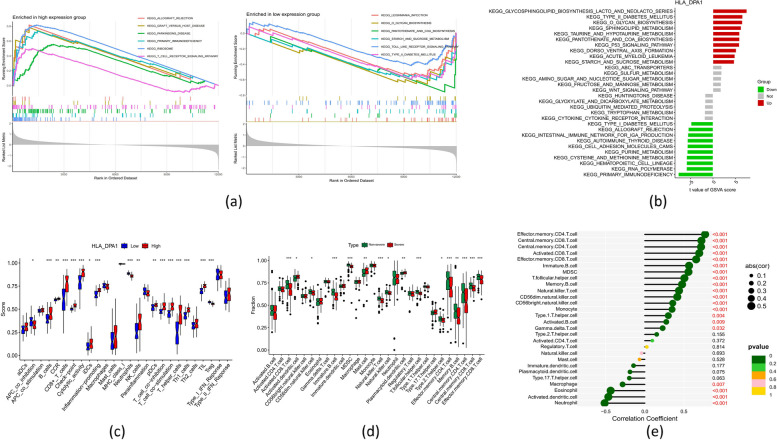
Functional analysis of HLA_DPA1. **a** Single-gene GSEA-KEGG pathway analysis in HLA_DPA1. **b** High- and low-expression groups based on the expression level of HLA_DPA1 with GSVA method. **c** The boxplots of the differences in immune cells infiltration between HLA_DPA1high and HLA_DPA1low groups. **d** The boxplots of the differences in immune cells infiltration between patients with severe and non-severe influenza. **e** Correlation analysis between HLA_DPA1 expression and the proportion of immune cells

In order to understand the possible functional importance of HLA_DPA1 in the pathogenesis of severe influenza, single-gene GSEA-KEGG pathway analysis was executed (Supplementary File 3), and the top six pathways enriched for HLA_DPA1 are presented in Fig. 10a. Overall, HLA_DPA1 was found to be involved in the pathological condition of severe influenza by regulating the immune or inflammatory responses such as KEGG_leishmania_infection, KEGG_Toll_like_receptor_signaling_pathway), carbohydrate and cofactor metabolism, and vitamin metabolism. The GSVA produced comparable outcomes (Fig. 10b).

### Analysis of infiltration of immune cells

Significance variances in the numbers of specific immune cell populations in whole blood samples from individuals with HLA_DPA1^low^ and HLA_DPA1^high^ were compared using ssGSEA. This approach revealed noteworthy suppressed adaptive immune responses in patients with HLA_DPA1^low^. This suppression was characterized by reduced levels of CD8 + T-cells, B-cells, two T-cell subsets (Th1-cells and Th2-cells), tumor-infiltrating lymphocytes (TIL), T-cell co-stimulation, antigen-presenting cell (APC) co-stimulation, as well as elevated levels of regulatory T-cells (Treg) and APC co-inhibition (Fig. 10c and Supplementary file 4). Similarly, suppressive adaptive immune responses were observed in patients with severe influenza, which manifested as decreased levels of key lymphocyte populations, including activated CD8 + T cells, B cells, CD4 + T cells, and memory CD8 + T cells, B cells, and CD4 + T cells (Fig. 10d). In addition, subsequent correlation data exhibited a remarkable positive association between the expression of HLA_DPA1 and the abundance of these lymphocytes (Fig. 10e and Supplementary File 5).

### Establishment of a key gene-based ceRNA network

**Fig. 11:**
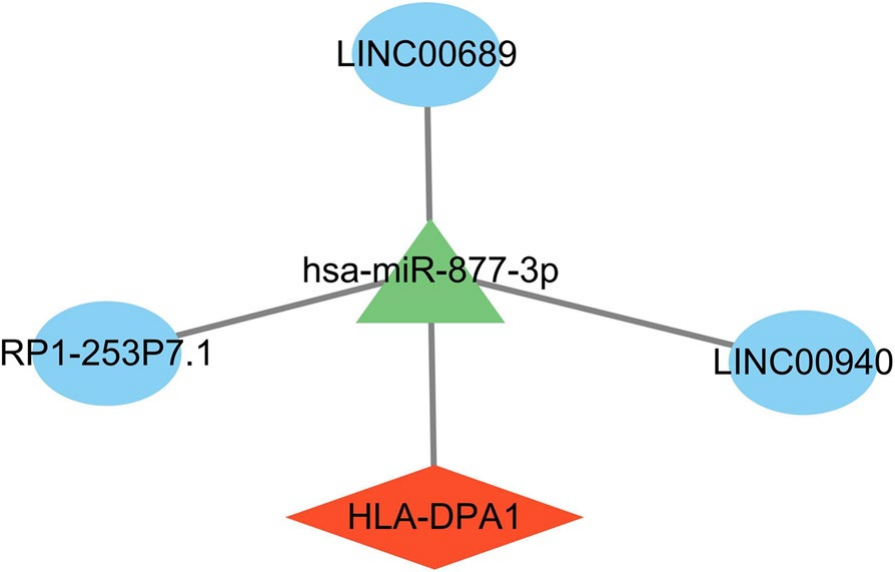
ceRNA network based on HLA_DPA1

A comprehensive analysis was executed by intersecting genes from the TargetScan, miRDB, and miRanda databases (Supplementary File 6) and via this approach, six miRNAs (hsa-miR-573, hsa-miR-1253, hsa-miR-877-3p, hsa-miR-429, hsa-miR-3182, and hsa-miR-22-5p) targeting HLA_DPA1 were screened. Based on starBase, three lncRNAs (LINC00689, LINC00940, and RP1-253P7.1) interacted with hsa-miR-877-3p. A ceRNA network comprising 5 nodes and 4 edges was established (Fig. 11).

### qRT-PCR

**Fig. 12:**
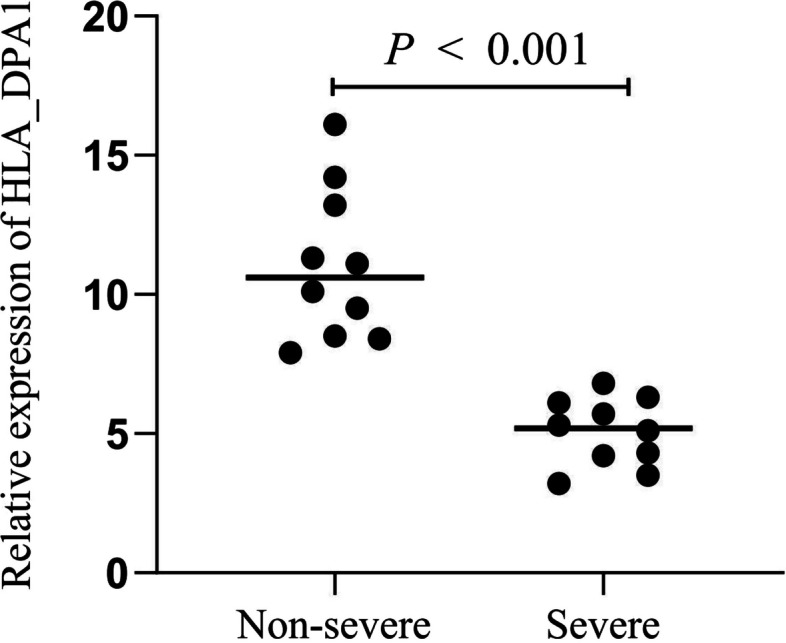
The mRNA levels of the HLA_DPA1 in blood samples from 10 pairs of severe and non-severe influenza patients

The mRNA levels of HLA_DPA1 in blood samples from patients afflicted with severe and non-severe conditions were verified using qRT-PCR. This showed a significant reduction in the expression of HLA_DPA1 in patients afflicted with severe influenza compared to those who remain non-severe by infection (Fig. 12).

## Discussion

Previous investigations have elucidated the host factors linked to the development of severe influenza. However, they have predominantly concentrated on a genetic event, genetic susceptibility [10–12]. Recently, transcriptomic investigations have documented comprehensive gene expression profiles pertaining to the host's response. The findings from these investigations suggest that the composition and functionality of gene sets deviate significantly among patients exhibiting different degrees of severity [13, 14]. Nonetheless, these findings were derived solely from a singular cohort study, thereby necessitating additional clinical validation and comprehensive functional analysis that needed to be explored. Thus, we have successfully recognized the key genes associated with severe influenza in the current study by integrating multiple datasets. Consequently, the outcomes obtained are anticipated to offer a more comprehensive understanding of the subject matter. Three distinct machine-learning methods were employed for the screening of potential key genes. The LASSO is a widely recognized regression analysis algorithm renowned for its distinctive variable selection and regularization features. These attributes are instrumental in mitigating the risk of overfitting and enhancing the accuracy of predictions [15]. The Support Vector Machine (SVM) is a well-established supervised machine learning approach that is commonly employed for classification and regression tasks. On the other hand, the Recursive Feature Elimination (RFE) algorithm is utilized to identify the most optimal combination of variables that maximizes the performance of the model [16]. Hence, the current investigation utilized the Support Vector Machine Recursive Feature Elimination (SVM-RFE) algorithm to ascertain feature biomarkers possessing exceptional discriminative capacity. The Random Forest technique is a widely used regression tree-based method that employs bootstrap aggregation and predictor randomization to attain notable predictive accuracy [17].

The candidate key genes obtained by overlapping the genes from the three algorithms exhibited higher reliability. Our study’s functional enrichment analysis displayed that DEGs between both influenza (severe and non-severe) cases were primarily associated with pathways with immune response and inflammation-related pathways. Moreover, the ICI analysis revealed a notable impairment in adaptive immune responses among patients afflicted with severe influenza, consistent with prior scientific findings [13, 18, 19]. Nguyen et al. [13] conducted a longitudinal study on patients hospitalized with acute influenza and found that a higher SOFA score was associated with lower adaptive-producing CD8 + T cell responses. Dunning et al. [18] reported that patients with the most severe illness exhibited a notable reduction in interferon (IFN)-related transcripts. The precise mechanisms responsible for inhibiting adaptive cellular immune responses during severe influenza infection remain poorly elucidated. The occurrence and progression of adaptive cellular immunosuppression may involve various mechanistic events, including directive killing, disruption of antigen presentation, apoptosis, abortive infection of primary human T cells, and T cell exhaustion or paralysis induced by viruses and cytokines [20–22].

From the candidate genes, HLA_DPA1 was selected as the key gene for patients with severe influenza requiring IMV, which showed the best differential performance in both the training and validation cohorts. Functional enrichment analysis suggested that HLA_DPA1 mainly participates in regulating immune and inflammatory pathways. HLA_DPA1 was significantly and positively associated with lymphocytes; thus, the patients with HLA_DPA1^low^ often showed deficient adaptive immunity and were more likely to be classified as critically ill. HLA_DPA1 is a major histocompatibility complex (MHC) class II-related gene [23]. HLA-DP-restricted T-cells and antimicrobial immune responses have also been identified [24, 25]. HLA-DPA1 polymorphism is a major determinant of hepatitis B virus clearance [26, 27]. A previous study reported that downregulation of HLA_DPA1 is associated with immunosuppression and increased mortality in sepsis [28–30]. In the context of severe infection, some inflammatory mediators are possibly involved in the down-regulation of the gene expression of MHC II [31–34]. For example, interleukin-10 (IL-10) can reduce the membrane expression of MHC II in monocytes. This reduction is attributed to the internalization and sequestration of mature MHC II molecules within the intracellular compartments [31, 32]. In an in vitro study, transforming growth factor-1 (TFG-1) downregulates MHC II mRNA expression by suppressing transcription factor class II transactivator (CIITA) mRNA transcription, while prostaglandin E2 was found to suppress MHC II mRNA expression in macrophages [33, 34]. The downregulation of MHC II leads to defective antigen processing, presentation, and as well as the proliferation of lymphocytes [35, 36]. The immunosuppressive state of the immune system significantly impedes the patient's ability to eliminate the primary influenza virus infection and enhances vulnerability to subsequent opportunistic infections, thereby resulting in many detrimental clinical outcomes in patients afflicted with influenza infection.

The present study has several noteworthy constraints. First, we must recognize the complex pathology of severe influenza, which is not driven by a single gene. Nevertheless, it can be asserted with a certain degree of certainty that the HLA_DPA1 gene exerts a pivotal influence on the progression of severe influenza and therefore merits prioritization in subsequent investigations. Second, the sample size was comparatively small despite our efforts to retrieve all the online data. Hub gene-encoding protein tests revealed a correlation between hub genes and disease severity. Furthermore, it is noted that the association between hub genes and immune cells is based on statistical correlation rather than establishing a causal relationship. Lastly, identifying DEGs in patients with both types of influenza has shed light on potential host factors associated with the chronicity of infection. However, the specificity of these factors to severe influenza infection has yet to be determined. Additional cell culture and animal studies are necessary to investigate these hub genes' roles and underlying mechanisms in severe influenza.

## Conclusions

In conclusion, the findings of our investigation declare that the HLA_DPA1 gene act as a crucial role in the immunopathological condition of severe influenza. Furthermore, because of the high discrimination potency and cost-efficient property of HLA_DPA1, its clinical assessment may provide an accurate and early diagnosis of severe influenza. Therefore, it is a promising candidate for targeted interventions for the management and prevention of severe influenza cases necessitating IMV.

## Materials and methods

### Data source

The National Center for Biotechnology Information (NCBI) Gene Expression Omnibus (GEO) database, accessible at (http://www.ncbi.nlm.nih.gov/geo) serves as a comprehensive repository for mRNA expression data pertaining to patients affected with influenza. The selection criteria employed in this study were as follows: I) Influenza infection was confirmed through the application of reverse transcription polymerase chain reaction (RT-PCR) methodology, which involved the analysis of respiratory tract samples; and ii) the disease severity classification was generally similar. In this investigation, the classification of severe influenza was established depending on the criterion of patients necessitating IMV; iii) Influenza patients were ≧ 16 years old, and intubated patients were included. Three datasets were obtained: GSE21802, GSE111368, and GSE101702. The GSE21802 microarray data consisted of blood samples obtained from 20 patients with severe influenza and 16 patients diagnosed with non-severe influenza, and the GSE111368 dataset comprised 69 samples of severe and 160 samples of non-severe influenza cases. The dataset GSE101702 included blood samples obtained from 107 individuals, consisting of 44 patients diagnosed with severe influenza and 63 with non-severe influenza. After the elimination of mRNA probes from the GSE21802 and GSE111368 datasets, the gene expression analysis was consolidated into a unified file, serving as the training dataset.

### Data processing and screening of differentially expressed genes

The integration of genomic data batches to increase statistical power is often hindered by batch effects or unwanted variation in data caused by differences in technical factors across batches. To remove the batch effect from different platforms and batches, the R sva package (https://bioconductor.org/packages/sva/) was employed to mitigate batch effects. Before conducting cross-platform normalization, the expression values of individual datasets underwent log2 transformation. Expression values obtained from various platforms or sample batches were subjected to normalization via the ComBat method. Principal component analysis was executed to validate the successful removal of batch effects. We used specific criteria to identify DEGs among both types of influenza (severe and non-severe) cases. The threshold points for selection were set at a significance *P* < 0.05 level and a minimum log fold change (logFC) > 1. The experimental findings were graphically represented using a volcano plot.

### Functional enrichment analyses

The enrichment analyses for Gene Ontology (GO) and Kyoto Encyclopedia of Genes and Genomes (KEGG) were executed via the R package 'clusterprofiler'. The significance threshold for these analyses was set with an adjusted FDR (false discovery rate) (FDR < 0.05) and *P*-value < 0.05. GO terms were categorized into three main classes: biological process (BP), molecular function (MF), and cellular component (CC). In this study, we presented the top 10 enriched terms.

### Candidate key genes identification

Three machine learning algorithms, least absolute shrinkage and selection operator (LASSO), Random Forest (RF), and Support Vector Machine (SVM), were utilized in this study to detect significant diagnostic genes for severe influenza. The LASSO is a regression analysis algorithm, which is characterized by variable selection and regularization. It helps avoid overfitting and improves the prediction accuracy. RF uses different independent decision trees to predict the classification or regression. The SVM is a supervised machine learning technique widely used in classification and regression. The recursive feature elimination (RFE) algorithm is employed to acquire the optimal combination of variables that maximizes the performance of the model. Therefore, this study utilized the SVM-RFE algorithm to identify potent biomarkers with superior discriminative ability. Thus the candidate genes will have higher reliability as they are identified by overlapping genes via three algorithms. To validate their expression levels in severe influenza samples, the dataset GSE101702 was utilized.

### Diagnostic performance examination

To assess the predictive efficiency of the candidate key genes for severe influenza, an ROC curve was plotted using the mRNA expression data obtained from patients diagnosed with influenza (severe and non-severe), sourced from both the training and validation datasets. The gene exhibiting the highest area under the ROC curve within the validation cohort was identified as a key gene.

Patients with key gene expression values above the median for all severe influenza patients were categorized as the gene^high^ group. In contrast, those with values below the median were assigned to the gene^low^ group. The differential expression of the key gene was determined using analysis of an unpaired t-test, with a significance level of *P* < 0.05. A fold change (FC, log2) threshold of > 0.5 or < -0.5 was also applied.

### Pathway evaluation by single-gene gene set enrichment analysis

The R GSEA package was utilized to conduct GSEA to identify the pathways linked to the key genes. This was achieved by assessing the correlations between the key genes and all other genes in the training dataset.

These genes were then ranked based on the strength of their correlative relationships. The “c2.cp.kegg.Hs.symbols” gene set was downloaded from the MSigDB database for GSEA analysis and an |NES|> 1, normalized *p*-value < 0.05, and FDR q-value < 0.25 denoted statistical significance. The genes were subsequently ranked according to the magnitude of their correlative associations. The gene set "c2.cp.kegg.Hs.symbols" was obtained from the Molecular Signatures Database (MSigDB) to conduct GSEA. Statistical significance was determined based on the criteria of an absolute Normalized Enrichment Score |NES|> 1, a normalized *p*-value < 0.05, and an FDR q-value < 0.25.

### Single-gene gene set variation analysis of key genes

The GSVA analyses of key genes were executed using the R GSVA package, with the KEGG pathway gene set as the background. Using the Limma package, a comparison of the GSVA scores for marker genes between the low- and high-expression groups was conducted. Significance variations between groups were evaluated via a threshold of |t|> 2 and a level of significance (*P* < 0.05). A positive value of t > 0 indicated pathway activation in the high-expression group, while a negative value of t < 0 indicated pathway activation in the low-expression group.

### Correlation between the key gene and infiltrating immune cells

The calculation of relative ICI levels in the training dataset was executed utilizing a ssGSEA algorithm. Immune cell enrichment levels were quantified using ssGSEA scores for each sample. Differential expression patterns of immune-infiltrating cells between the key gene^high^ and key gene^low^ groups, and patients with both cases of influenza (severe and non-severe), were monitored via violin plots. The Spearman correlations between ICI and the key gene were assessed via the 'ggplot2' package in the R programming language.

### Development of ceRNA network

The identification of miRNAs that interact with key genes was performed using the StarBase computational tool. The mRNA sequences of these genes were obtained from NCBI. Human miRNA sequences were acquired from miRbase. Subsequently, the TargetScan, miRDB, and miRanda databases were employed to forecast the target genes of miRNA. StarBase was used to conduct screening for interactions between mRNA-lncRNA. This facilitated the establishment of a comprehensive network involving mRNA, microRNA (miRNA), and lncRNA.

### qRT-PCR

Total RNA content was extracted from a set of 10 paired severe and non-severe influenza samples by the reagent of TRIzol (Life Technologies, Carlsbad, CA, USA) as per the manufacturer's protocol guidelines. The reverse transcription process was executed via PrimeScript RT Master Mix (Takara in Tokyo, Japan). The resulting cDNA was amplified using the ABI 7700 system (Applied Biosystems in CA, USA). β-lactin was employed as housekeeping control to evaluate the relative expression levels. It was assessed by utilizing the 2-ΔΔCt method. The following primer sequences were used for the qRT-PCR:

- Forward: 5’-CTGCCCAGAACAGATTACAGC-3’,
- Reverse: 5’-ACAGTCTCCGTTGTCTCAGG-3’

### Data analysis

The statistical analyses were executed by applying R software (version 4.2.0). Statistical analysis was performed using an unpaired t-test for variables that revealed a normal distribution. At the same time, the Mann–Whitney U test was utilized for variables that displayed a non-normal distribution. Spearman's correlation coefficient was employed to conduct the correlation analysis. Statistical significance was determined by assessing differences with a *p* < 0.05.

### Supplementary Information

**Supplementary Material 1.** Detailed results of GO analysis.

**Supplementary Material 2.** Detailed results of KEGG analysis.

**Supplementary Material 3.** Detailed results of single-gene GSEA-KEGG pathway analysis for HLA_DPA1.

**Supplementary Material 4.** Detailed results of ssGSEA for individuals with HLA_DPA1^low^ and HLA_DPA1^high^.

**Supplementary Material 5.** Correlation analysis of HLA_DPA1 expression level with the abundance of lymphocytes

**Supplementary Material 6.** Predicted genes from the TargetScan, miRDB, and miRanda databases.

## Data Availability

Publicly available datasets were analyzed in this study. These data can be found in GSE111368 (https://www.ncbi.nlm.nih.gov/geo/query/acc.cgi?acc=GSE111368), GSE101702 (https://www.ncbi.nlm.nih.gov/geo/query/acc.cgi?acc=GSE101702), GSE70866 (https://www.ncbi.nlm.nih.gov/geo/query/acc.cgi?acc=GSE70866) and GSE10667 (https://www.ncbi.nlm.nih.gov/geo/query/acc.cgi).

## Abbreviations

GEO: Gene Expression Omnibus
DEGs: Differentially Expressed Genes
LASSO: Least Absolute Shrinkage and Selection Operator
SVM-RFE: Random Forest, and Support Vector Machine-Recursive Feature Elimination
ssGSEA: Single-sample Gene Set Enrichment Analysis
GSVA: Gene Set Variation Analysis
ROC: Receiver Operating Characteristic Curve
AUC: Area Under the Curve
